# Fast antibody responses by immuno-targeting and nanotechnology strategies versus HBsAg vaccine

**DOI:** 10.22038/IJBMS.2021.52715.11896

**Published:** 2021-04

**Authors:** Mahsa Rezaei, Seyed Nezamedin Hosseini, Ramazan Ali Khavari-Nejad, Farhood Najafi, Mehdi Mahdavi

**Affiliations:** 1 Department of Biology, Sciences and Research Branch, Islamic Azad University, Tehran, Iran; 2 Department of Hepatitis B Vaccine Production, Production and Research Complex, Pasteur Institute of Iran, Tehran, Iran; 3 Department of Resin and Additives, Institute for Color Science and Technology, Tehran, Iran; 4 Recombinant Vaccine Research Center, Tehran University of Medical Sciences, Tehran, Iran; 5 Immunotherapy Group, The Institute of Pharmaceutical Sciences (TIPS), Tehran University of Medical Sciences, Tehran, Iran; 6 Department of Immunology, Pasteur Institute of Iran, Tehran, Iran

**Keywords:** Avidity Index, Hepatitis B, Immune response, Iron oxide nanoparticle, Mannose

## Abstract

**Objective(s):**

Though immunization with HBsAg has been routine since the 1980s, it has numerous limitations such as low or none humoral immune responses. Today, nanotechnology is used in vaccinology to achieve higher potency. The present study deals with the achievement of fast antibody response of humoral immune responses using immune-targeting through mannosylated nanocarriers of the vaccine.

**Materials and Methods:**

Mannose sugar and HBsAg were attached to the surface of iron oxide nanoparticles. Mannosylated iron oxide nanoparticles conjugated HBsAg (HBsAg +MLCMNP), iron oxide nanoparticles conjugated HBsAg (HBsAg +LCMNP), hepatitis B vaccine, and mere HBsAg were injected twice to BALB/c mice subcutaneously, while suitable control groups were considered. Specific total IgG antibodies were evaluated on the 7th and 14th days after the final immunization. The avidity maturation of the humoral immune response was assessed with an optimized ELISA. Graph pad prism software was used to analyze statistical data.

**Results:**

Results showed that on the seventh day of the final shooting, the mannosylated nano-vaccine caused higher antibody response induction than nano-vaccine without mannose and commercial vaccine groups. After 14 days of the second injection, a significant difference was seen versus the nano-vaccine without mannose but not the commercial vaccine group. In addition, the avidity index in mannosylated nano-vaccine showed a significant increase compared with the nano-vaccine without mannose and mere HBsAg group but not compared with the commercial vaccine.

**Conclusion:**

It seems that mannosylated nano-vaccine has more potency to achieve fast antibody responses and also higher quality of humoral immune response.

## Introduction

Human Hepatitis B Virus (HBV) is a member of the *Hepadnaviridae* family (1, 2) and can cause a liver infection that may lead to acute hepatitis or chronic hepatitis, cirrhosis, and hepatocellular carcinoma (3, 4). 

The hepatitis B vaccine that is used now is HBsAg formulated in alum adjuvant. The final immunization of hepatitis B vaccine leads to the promotion of humoral immune response and neutralizing antibody and reduces the rate of hepatitis B infection (5-7).

Approximately 10% of the vaccinated people display poor responses and are considered non-responders and impose a large economical force on the healthcare system due to their susceptibility to chronic infections (8). So, introduction of novel formulations of the HBsAg vaccine to improve their potency in the induction of cell-mediated and humoral immune responses seems to be useful and efficient (9, 10).

Aluminum salt known as alum is a strong Th2 stimulator that is usually used as an adjuvant to enhance the immunogenicity of HBsAg but it poorly stimulates Th1 immunity and is considered a standard adjuvant which has been approved for human use (11) and was the first adjuvant used in human vaccines (10). The common preventive vaccine against HBV does not induce protection in certain groups such as dialysis patients, newborns of mothers who are hepatitis B carriers, and even in some healthy people (12). In recent years, nano-particulate carriers have become more prominent due to their size and ability of controlled delivery of bioactive materials in the technology of drug delivery and vaccines (13). Among the nano-carriers, inorganic adjuvants commonly facilitate the delivery of antigens allowing them to be released in a sustained manner, increase immunogenicity, render antigens efficiently to specific purposes, and stimulate a specific immune response. Inorganic nanomaterials can also modulate the immune responses to the desired type and enhance the efficacy of vaccines (14). Among inorganic adjuvants, iron oxide nanoparticles have special importance in the field of vaccines because of their unique properties (non-toxicity, easy to synthesize in controllable sizes, adjustable surface charge). These particles can be coated with polymers and inorganic materials like silica to keep them stable and can be attached to different biomaterials such as antigens, proteins, and antibodies for medical applications especially vaccines (15-17). However, most nanocarriers fail to deliver biological agents to the target areas in the body due to the non-specific absorption of nanocarriers to the immune system and their poor targeting (13). In this regard, the covalent bonding or physical absorption of site-specific ligands for nanocarriers can be a suitable and targeted strategy for inducing immune responses in the vaccines and improving the delivery of drugs to the target organs (18). In various studies, it has been shown that if a ligand covalently binds to a nanocarrier, it can work more efficiently and increase the carrier’s potential to reach the targets faster (19). The abundance of mannose receptors on macrophages and dendritic cells has attracted the attention of researchers to use this inexpensive and available sugar for targeting nanocarriers (20, 21). Targeting antigens to mannose receptors on antigen-presenting cells such as macrophages and dendritic cells has facilitated the entry, processing, and delivery of antigens through both MHC class I and class II and thus has increased immune responses (22, 23). 

Here, we investigated antibody responses and avidity index of antibody response in the experimental mice immunized with a novel vaccine in which iron oxide nanoparticles were incorporated by both mannose and hepatitis B surface antigen (HBsAg). This novel vaccine as delivery vehicle is targeted to dendritic cells and macrophages to increase the vaccine potency. 

## Materials and Methods

***HBV vaccine and HBsAg***

HBsAg and commercial HBsAg vaccines (alum-based vaccine) were procured from Dr. Hosseini from the Department of Hepatitis B Vaccine Production, Pasteur Institute of Iran (Karaj, Iran). Nano-vaccines (Iron nanoparticles with HBsAg and mannose (HBsAg-MLCMNP) and iron nanoparticles with HBsAg (HBsAg-LCMNP)) were prepared using chemical reactions as reported previously(24). Briefly, iron oxide nanoparticles with HBsAg and mannose and iron oxide nanoparticles with HBsAg were provided by the co-precipitation procedure and chemically were linked to HBsAg and mannose.

Nano-vaccines’ physicochemical characteristics including zeta potential, size, efficiency of antigen and mannose-binding, structure of HBsAg bonded nanoparticles, toxicity on HEK293 cell line, *in vitro* release of nanoparticles, uptake of nano-vaccine by macrophage/dendritic cells, and also cytokines gene expression profile of macrophage/dendritic cells exposed to these nano-vaccines were assessed in the previous report (24).

***Experimental animals***

***Achievement***

Female BALB /c mice (6–8 weeks old) were purchased from the Pasteur Institute of Iran (Karaj, Iran) and kept in animal care centers under standard conditions. All mice were kept for seven days before the start of the trials with access to water, food, and observing standard conditions including equal light/darkness and room temperature (20 to 22 °C). All trials on animals were performed based on the Animal Care Protocol of Pasteur Institute of Iran.

***Immunization and study planning***

The mice of experimental groups were divided into seven groups (Table 1). While group 1 and group 2 were administrated the vaccine formulated with nano-vaccine bonded to mannose and HBsAg and nanovaccine without mannose, respectively, group 3 was immunized with the commercial HBsAg-Alum and Group 4 with HBsAg alone. Mice were injected subcutaneously on days 0 and 14. Also, control groups were included iron oxide nanoparticles (MNP), iron oxide nanoparticles-Mannose (MNP-Mannose), and PBS. All vaccine formulations consisted of 5 µg of HBsAg. The last immunization was done. After the first and then the second-week total antibodies were measured by ELISA, and the avidity index was calculated by treating serum with NH_4_SCN in the ELISA reader.

***Specific total IgG response***

Specific total IgG antibodies were assessed using an optimized indirect ELISA method on the sera samples obtained at the 7^th^ and 14^th^ days after the last immunization. Briefly, wells of 96-well ELISA Maxisorp plates (Nunc, Naperville, IL) were coated with 100 µl of HBsAg (5 µg/ml) in PBS and incubated overnight at 4 °C. The wells were washed with washing buffer (PBS containing 0.05% Tween20) 3 times and blocked with 5% skimmed milk in washing buffer (blocking buffer) for 1 hr at 37 °C. Serial dilutions of sera (dilutions of 1/10 to 1/1280 of sera 7^th^ day after the last injection and dilutions of 1/25 to 1/819200 of sera on the 14^th^ day after the last injection) were prepared in washing buffer containing BSA1%. After washing the wells (five times), 100 µl of each dilution was poured into each well in duplicate and followed by incubation at 37 °C for 2 hr. Then the wells were washed with washing buffer (five times) and 100 µl of 1/10000 dilution of anti-mouse conjugated to horseradish peroxidase (HRP) (Sigma, USA) in washing buffer plus BSA1% added to the wells and incubated for 2 hr at 37 °C. Later, the wells were washed five times with washing buffer and TMB substrate (100 µl) was added and incubated for 30 min in the dark site. The reaction was stopped by adding 100 µl of 2N H_2_SO_4_ and color density was read at A_450/630 nm _with an ELISA plate reader (AWARENESS, USA). 

***Avidity index of the humoral immune response***

Antibody avidity index was measured by a routine protocol that was set up in our laboratory using thiocyanate (NH_4_SCN) as the chaotropic agent (25). In brief, 100 µl of HBsAg (5 µg/ml) were coated in wells of 96-well ELISA Maxisorp plates (Nunc, Naperville, IL). All sera were diluted 1:800 (optimized dilution) and added to the plates in two different sets. The plates were incubated (37 ºC for 2 hr) and after that were washed five times with washing buffer. After washing, some plates were treated with 100 µl of NH_4_SCN 1.5 M, and the rest were treated with 100 µl of PBS (10 min at 37 °C). Then washing buffer was used for washing all plates (five times) and plates were incubated with 100 µl of anti-mouse IgG-horseradish peroxidase for 60 min at 37 °C. After final washing, the TMB substrate was added to each well for 30 min and then 100 µl 2NH2SO4 was used for stopping and the plates read at 450 nm by ELISA plate reader (AWARENESS, USA). In order to calculate the avidity index, NH_4_SCN treated plates at OD450 nm were divided into PBS treated plates at OD450 nm (26). 

***Statistical analysis***

Results were shown as Mean±SD. The analyses of data were performed using Graph pad prism (V 6.01) software package. THE two-way ANOVA method was applied to analyze the difference between experimental groups. *P*-value=0.05 was considered to show statistically significant differences. 

## Results

***Results of specific IgG response***

**Figure 1:**
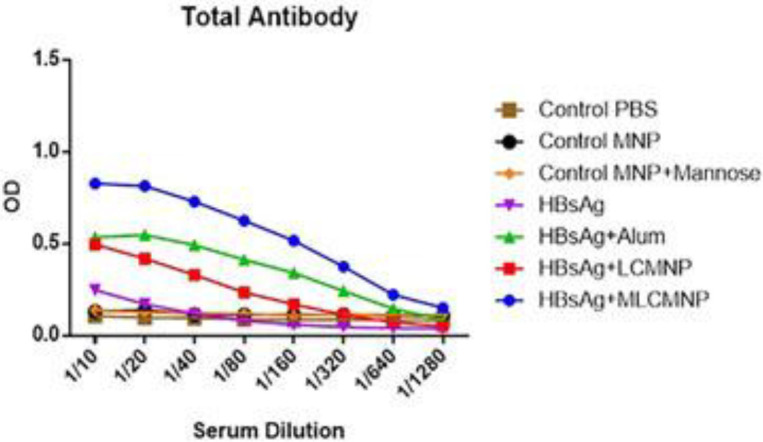
Specific total IgG responses, one week after the last vaccination. Mannosylated nanovaccine enhanced antibody responses versus mannose-free nano-vaccine group and also commercial alum-based vaccine

One week after the last immunization results of total IgG antibodies (Figure 1) showed that immunization with mannosylated iron oxide nanoparticles conjugated HBsAg (HBsAg- MLCMNP) (at dilutions of 1/10 up to 1/320), iron oxide nanoparticles conjugated HBsAg (HBsAg-LCMNP) (at dilutions of 1/10 up to 1/40), and commercial HBsAg-Alum vaccine (at dilutions of 1/10 up to 1/80) enhanced total IgG antibodies compared with control groups (*P*<0.0363). Immunization with mannosylated iron oxide nanoparticles conjugated HBsAg showed a significant enhancement (at dilution 1/10) versus commercial HBsAg- Alum group (*P*=0.0363). Moreover, the addition of mannose to the formulation of nano vaccine in mannosylated iron oxide nanoparticles conjugated HBsAg group resulted in a significant enhancement (at dilutions of 1/10 up to 1/320) versus iron oxide nanoparticles conjugated HBsAg group (*P*<0.0363).

**Figure 2:**
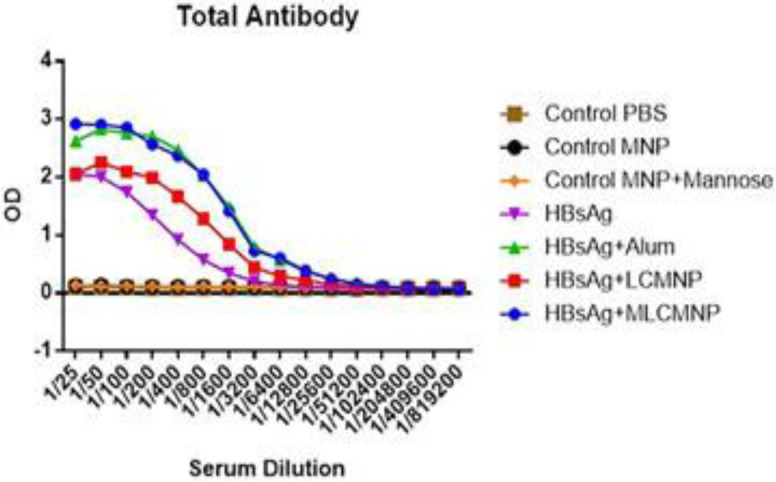
Specific total IgG responses, two weeks after the final vaccination. Conjugation of nanovaccine with mannose enhanced antibody responses versus mannose-free nano-vaccine. In addition, mannosylated nano-vaccine shows comparable total IgG response to commercial HBsAg vaccine

Two weeks after the final immunization results of total IgG antibodies (Figure 2) showed that immunization with mannosylated iron oxide nanoparticles conjugated HBsAg (at dilutions of 1/25 up to 1/3200), iron oxide nanoparticles conjugated HBsAg (at dilutions of 1/25 up to 1/1600), commercial HBsAg-Alum vaccine (at dilutions of 1/25 up to 1/3200) enhanced total IgG antibodies compared with control groups (*P*<0.05). Immunization with mannosylated iron oxide nanoparticles conjugated HBsAg and commercial HBsAg-Alum group showed no significant difference between their responses. The addition of mannose to the formulation of nano vaccine in mannosylated iron oxide nanoparticles conjugated HBsAg group also resulted in a significant increase (at dilutions of 1/25 up to 1/1600) versus iron oxide nanoparticles conjugated HBsAg group (*P*=0.0378).

***Results of avidity index***

**Figure 3:**
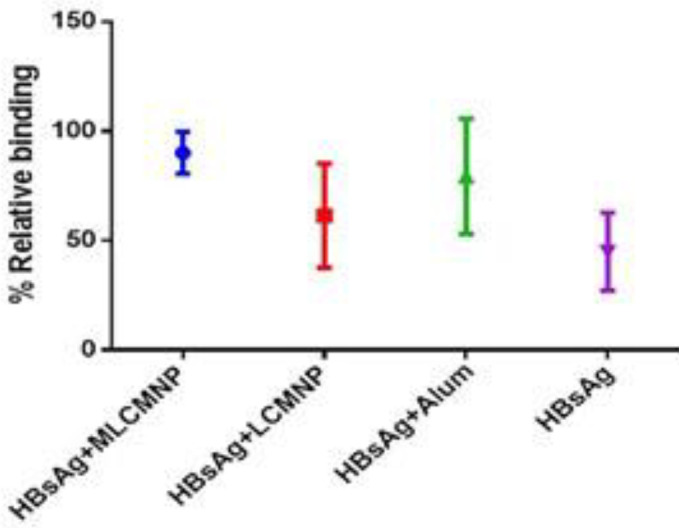
Relative avidity index of antibody response of experimental groups. The avidity of the serum was calculated by its ability to remain connected to HBsAg in the attendance of NH_4_SCN. Avidity indexes were assessed by an optimized ELISA reader, after immunization of mice with HBsAg alone, commercial HBsAg vaccine, HBsAg +MLCMNP, and HBsAg+ LCMNP. Mannosylation of nanovaccine improved avidity index versus nanovaccine without mannose and also about 11% higher than the commercial alum-based vaccine

The avidity of the antisera was measured to assess antibody affinity maturation through the displacement method using 1.5 M NH_4_SCN as the chaotropic agent (25). Although all sera samples tested had high relative avidity indices (RAI), RAI was significantly lower in those immunized groups with mere HBsAg (44.95%). Immunization with MLCMNP-HBsAg elicited antibodies with higher RAI than HBsAg (*P*=0.0001) and LCMNP-HBsAg groups (*P*=0.007). Comparing the two adjuvants, sera from mice immunized with MLCMNP-HBsAg had about 11% higher avidity than mice immunized with HBsAg-alum (mean= 90.10% and 79.29%, respectively), though there was no significant difference between the two groups (Figure 3). 

**Table 1:** Experimental groups (Number of mice) and vaccine formulations

**Groups**	**Vaccine formulations**	**Number**
1	MLCMNP+HBsAg	9
2	LCMNP+HBsAg	9
3	Commercial HBs vaccine	9
4	HBsAg	9
5	Control MLCMNP	5
6	Control MNP	5
7	Control PBS	5

## Discussion

Recently the use of nanoparticles as vaccine adjuvants and carriers has attracted the attention of researchers (27). The iron oxide nanoparticles have been applied, due to their unique properties, in different fields such as science and technology, (28-31). They are also used in biomedical applications such as vaccines (16, 32) because of their size compatibility to cells, genes, and viruses (33). Not only does the coating of iron oxide nanoparticles ensure the stability of nanoparticles in solution polymers and inorganic materials but also binds to several biological ligands needed for medical applications (34). The active targeting of vaccine antigens to the cell surface receptors on dendritic cells and macrophages can potentially enhance the efficacy of such vaccines in inducing immune responses. One of the desired receptors in this field is the mannose receptor (35-37). Mannose receptors are present on the surface of dendritic cells and macrophages at high density that are involved in entering, processing, and presentation of antigens through both MHC class I and MHC class II presentation pathways and also triggering cell signaling (23, 38-40). Therefore, the targeting of the mannose receptor can be a promising approach to improve the immunogenicity of vaccine candidates. 

In this study, the results of specific total IgG antibodies showed that one week after the last vaccination the mannosylated nano-vaccine was more successful than mannose-free nano-vaccine in inducing specific antibody responses and in comparison with commercial vaccines and even mere HBsAg. Two weeks after the last immunization, the antibody response to the commercial vaccine was increased but not significantly. This increase demonstrated fast antibody responses in the mannosylated nano-vaccine group. So, potentially it is expected that this strategy can induce fast protection in vaccine recipients. These results are consistent with the results of previous studies (41, 42). It has been shown that many vaccines effectively deliver antigens to receptors and improved immunogenicity (43-45) based on nanomaterials. The type of bond of any adjuvant is very effective in enhancing the immune response. Previous results have shown that both physical adsorption and chemical conjugation of mannose into nanoparticles results in enhancing the absorption of nanoparticles by dendritic cells. MPL has a physical bond with alum (in Fendrix vaccine) and stimulates toll-like receptors where antigens are bond with antigen-presenting cells (22, 46). In this study, the chemical bond between antigen and nanoparticles and mannose sugar makes the delivery of antigen and the binding of mannose sugar to mannose receptors better and therefore promotes better humoral immune responses. The entering of targeted nanoparticles into the mannose receptors is faster than their entering into the scavenger receptors (47). This may be due to the purposeful and effective presentation of antigen to dendritic cells, which stimulates T-cells and consequently, cytokines released from T-cells. The cytokines help B cells to differentiate into plasma cells and produce a higher level of antibodies (48). 

Antibody avidity rises over time when encountering an antigen. Memory responses are defined by generation of high-avidity antibodies; thus, avidity could be considered a substitute for successful priming (49). In this study nano-adjuvant without mannose showed a lower avidity index versus alum-based vaccine but when mannose was bonded to the vaccine nano-formulation avidity index increased about 11 percent versus alum-based vaccine. This increase in quantity showed the performance of mannose in the nano-structure in empowering of antibody quality. In a study, it has been shown that nano-vaccine induces rapid and long-lived protection against experimental infection of pneumonic plague (50). Besides, it has been demonstrated that nano-vaccine chemistry and composition can trigger antibody response with higher maturity and long duration (51).

## Conclusion

In general, the results of this study showed that iron oxide nanoparticles can be a good carrier for hepatitis B antigen and the utilization of mannose in this structure can stimulate fast antibody response with higher quality of antibody response. 
